# Stephen M. Levin: 1941–2012

**DOI:** 10.1289/ehp.120-a186

**Published:** 2012-05-01

Stephen M. Levin died as a result of cancer on 7 February 2012. He was born to Sam and Sarah Levin of Philadelphia, Pennsylvania, on 16 October 1941. He graduated from Wesleyan University in 1963 and earned his medical degree from New York University School of Medicine in 1967. After completing a surgery internship and a psychiatry residency, he returned to Pennsylvania as a general practitioner. In 1979 he joined the Irving J. Selikoff Center for Occupational and Environmental Medicine at Mount Sinai School of Medicine in New York City.

Levin, a professor of occupational medicine in the Department of Preventive Medicine of the Mount Sinai School of Medicine, served as a consultant to the New Jersey, New York State, and New York City departments of health. His primary research interests were asbestos-related disease and other occupational lung diseases, and heavy metal toxicity. For almost 20 years Levin served as the co-director of the Selikoff Center, leading it to prominence in medical care of workers exposed to occupational hazards. He excelled at using the center’s research as evidence to advocate for policies that protect workers’ health.

Levin’s leadership came to be tested on 11 September 2001. Soon after the World Trade Center towers fell, Levin organized his team to help protect thousands of workers who descended on the site of the disaster to help victims and conduct the recovery effort. On the day after the disaster, Levin was already in touch with labor unions to implement worker protections, even though authorities had not yet warned of environmental hazards.

Levin made it his mission to advocate for the creation of the World Trade Center Health Program, a model program in disaster response that currently cares for nearly 30,000 of the women and men who worked at the site. He fiercely petitioned for more services for affected workers because he foresaw that they would need long-term medical care as a result of their exposures. At a memorial in Levin’s honor, James Grogan, the president of the International Association of Heat and Frost Insulators and Allied Workers, stated, “Wherever there was something to be done for working men and women, you found Dr. Steve’s fingerprint on it.”

Recently, one of Levin’s battles on behalf of workers was won: The James Zadroga 9/11 Health and Compensation Act was amended to include cancer as one of the health conditions covered in the program. According to Carolyn B. Maloney, congresswoman representing New York’s 14th District, “If it weren’t for Dr. Steve, this bill would not have passed.”

Levin had more recently expanded his work to help residents of Libby, Montana, where vermiculite, a fibrous material similar to asbestos, has affected community residents and miners. “He helped thousands who have purpose but not resources,” said Brad Black, a physician working with the Libby community.

Philip J. Landrigan of the Mount Sinai School of Medicine said of Levin, “Steve was a doctors’ doctor, a mentor, and a hero.”

